# High MMP14 and low decorin expression correlate with disease progression and metastasis in Brazilian prostate cancer patients

**DOI:** 10.1007/s10735-026-10869-1

**Published:** 2026-06-25

**Authors:** Amanda L. Francelino, Fernando Terzioti, Glauco A. F. Vitiello, Phelipe Oliveira Favaron, André L. L. Vanzela, Alda F. M. L. Guembarovski, Carlos A. Miqueloto, Karen B. de Oliveira, Roberta L. Guembarovski

**Affiliations:** 1 Laboratory of Mutagenesis and Oncogenetics, Department of General Biology, State University of Londrina, Celso Garcia Cid Highway, PR-445, Km 380, Londrina, PR 86057-970 Brazil; 2 Londrina Cancer Hospital, Londrina, PR Brazil; 3 Department of Immunology, Parasitology and General Pathology, State University of Londrina, Londrina, PR Brazil; 4 Laboratory of Extracellular Matrix, Department of General Biology, State University of Londrina, Londrina, PR Brazil; 5 Laboratory of Cytogenetics and Plant Diversity, Department of General Biology, State University of Londrina, Londrina, PR Brazil; 6 Micropar Private Laboratory of Histopathological Analysis, Londrina, PR Brazil; 7 Laboratory of Molecular Genetics and Immunology, Department of Pathological Sciences, State University of Londrina, Londrina, PR Brazil

**Keywords:** Immunohistochemistry, Tumor dissemination, Prognosis, ECM

## Abstract

Prostate cancer (PCa) remains a highly prevalent malignancy and a leading cause of cancer-related mortality, frequently attributed to late-stage diagnosis. Matrix metalloproteinase 14 (MMP14) and Decorin (DCN) are key extracellular matrix (ECM) components essential for tissue remodeling and tumor suppression. This study evaluated the immunohistochemical expression of MMP14 and DCN in relation to clinicopathological parameters in PCa tissue samples (*n* = 92), specifically examining the malignant epithelium, adjacent non-tumor tissue, and peritumoral stroma. High MMP14 expression in tumor cells was predominant in metastatic patients and significantly associated with the presence of metastases (*p* = 0.012), with overexpression observed in 60% of these cases. Conversely, diminished DCN staining in the peritumoral ECM was linked to metastatic disease (63% low expression). Furthermore, a significant correlation was identified between low stromal DCN expression and ISUP grades 4–5 (*p* = 0.047; 66% of high-grade cases). Multivariate analysis confirmed that MMP14 expression profile serves as an independent predictor of metastasis (*p* = 0.01). In conclusion, our findings suggest that ECM remodeling, driven by dysregulation of MMP14 and DCN, plays a pivotal role in PCa progression, highlighting their potential as prognostic biomarkers for patient stratification and therapeutic guidance.

## Introduction

Prostate cancer (PCa) has a high incidence worldwide and is the most common type of malignant tumor among men (Sung et al. 2021; American Cancer Society 2025a). Its etiology is multifactorial, and development occurs slowly, with symptoms generally appearing only in advanced stages of the disease, which leads to late diagnoses, especially after the age of 65 years (Rego et al. 2020).

Detection methods used for PCa screening include digital rectal examination and detection of serum levels of prostate-specific antigen (PSA), a protein found in the blood that is used as a biomarker for changes in the prostate (American Cancer Society 2025b). However, PSA has low specificity for cancer, since men with levels considered normal (< 4 ng/ml) can develop high-grade malignant tumors (Lin 2009), highlighting the need for new markers.

Mortality rates associated with PCa increase significantly with factors such as advanced age, the presence of comorbidities, and tumor progression to advanced stages (Braga et al. 2021), including the development of metastases. Due to the complexity and incomplete understanding of the metastatic cascade, identifying highly aggressive phenotypes—characterized by rapid cell proliferation and migration into adjacent tissues—remains a challenge (Sahai 2007). While it is established that the bones and lungs are the primary metastatic sites for PCa, the underlying mechanisms remain poorly defined (Bubendorf et al. 2000). Therefore, advancing the knowledge of the disease’s most aggressive forms is essential for identifying new therapeutic targets and developing more effective treatments (Ponte et al. 2021).

In this context, Matrix Metalloproteinase 14 (MMP14) and the proteoglycan Decorin (DCN) are key components of the extracellular matrix (ECM) involved in remodeling processes and cell signaling pathways (Souza and Pinhal 2011; Niland et al. 2025) These molecules participate in events that promote tumorigenesis and cell migration, potentially influencing the metastatic cascade (Pach et al. 2021; Diehl et al. 2021).

A recent study observed that high expression of MMP14 in the ECM of the tumor microenvironment interferes with the progression to stage 3 colorectal cancer, due to the action of cancer associated fibroblasts that increase the expression of the protein, facilitating tumor progression (Makutani et al. 2022). It was also observed that DCN acts by modulating mechanisms that inhibit growth, cell proliferation and angiogenesis due to its ability to interact with cell receptors through signal transduction, with the main function of inducing signaling cascades that suppress tumorigenesis (Diehl et al. 2021). Thus, these are molecules that may be associated with the development of more advanced stages of PCa (Suhovskih et al. 2013; Tan et al. 2023).

Once a tumor is detected, the diagnosis is established through histopathological analysis of tissue fragments obtained via biopsy (Nascimento et al. 2022). This procedure enables the morphological characterization of the lesion and determines the tumor grade, facilitating the definition of more specific and individualized treatment strategies. Furthermore, tissue immunostaining has emerged as a crucial tool in clinical oncology, as it can reveal altered expression patterns of key molecules, highlighting their potential as promising tumor biomarkers (Mebratie and Dagnaw 2024).

In Brazil, the Unified Health System (SUS) manages a substantial volume of cancer screenings and oncological care. Data from the SUS database (TABNET/DATASUS) for 2025 reveal that among 18,304 men diagnosed with PCa, 46.2% presented with advanced-stage disease. Given this clinical landscape, investigating molecular biomarkers in patients treated within the SUS framework is of significant public health relevance. Furthermore, immunohistochemistry remains a cornerstone in oncology for supplementing diagnosis and guiding therapeutic management. Therefore, this study aimed to evaluate the immunohistochemical profiles of MMP14 and DCN in PCa samples from a Brazilian cohort, analyzing their correlation with established clinicopathological prognostic parameters across different risk groups.

## Materials and methods

### Patient cohort and sample selection

This study was approved by the Institutional Review Board (Research Ethics Committee Involving Human Beings) of the State University of Londrina (under protocol number CAAE: 88883625.5.0000.5231). All participants provided written informed consent prior to inclusion, and the study was conducted in accordance with the principles of the Declaration of Helsinki.

A total of 92 formalin-fixed paraffin-embedded (FFPE) tissue samples were obtained from Brazilian patients diagnosed with PCa. These patients were treated through the Brazilian Unified Health System (SUS) at the Londrina Cancer Hospital (Londrina, Paraná, Brazil). Based on clinical and pathological data — including PSA levels, ISUP grade, and the TNM staging system—patients were categorized into risk groups according to the National Comprehensive Cancer Network guidelines (NCCN Version 1.2023): low-risk (LRG, *n* = 11), intermediate-risk (IRG, *n* = 21), high-risk (HRG, *n* = 32), and metastatic (M, *n* = 28) (Table 1).

Although the TNM system consists of three independent parameters, a single assigned TNM value was used to subclassify patients. Patients were stratified into four risk groups as follows: low-risk localized disease (LRG) was defined by PSA < 10 ng/mL, ISUP grade 1, and clinical stage T1–T2a; intermediate-risk localized disease (IRG) included patients with PSA 10–20 ng/mL, ISUP grade 2–3, or stage T2b–c; and high-risk localized disease (HRG) comprised patients with PSA > 20 ng/mL, ISUP grade 4–5, or stage T3. The metastatic group (M) included patients with any PSA value or ISUP grade presenting with T/N/M1 staging. All samples corresponded to the primary malignant tumor, and only treatment-naïve patients (no prior chemotherapy and/or radiotherapy) were included in the study.

**Table 1:** Demographic data relating to the sample group of 92 patients

Parameters	No of individuals
Age	
Average	68.14 years (8.110892)
≤ 64 years	28
≥ 65 years	56
Risk groups	
Low risk (LRG)	11
Intermediate risk (IRG)	21
High risk (HRG)	32
Metastatic	28

### Immunohistochemical analysis of MMP14 and DCN

Of the 92 samples, FFPE blocks were evaluated by a pathologist using hematoxylin and eosin (H&E) staining to identify the presence of malignant tissue, adjacent non-tumor tissue, and peritumoral ECM. Representative blocks were selected and sectioned at a thickness of 6 μm. The sections were then mounted on silanized slides (Starfrost, Knittel, Germany).

Immunostaining was performed following the protocol described by Pereira et al. (2023), with modifications to the antigen retrieval step (heat cycles: 5 min at 80 W, followed by two 6-min cycles at 60 W). Sections were incubated overnight at 4 °C in a humidified chamber with rabbit polyclonal anti-MMP14 [LF-17] and anti-DCN [LF-114] antibodies (1:200, v:v), both provided by the National Institute of Dental and Craniofacial Research.

**Fig. 1:**
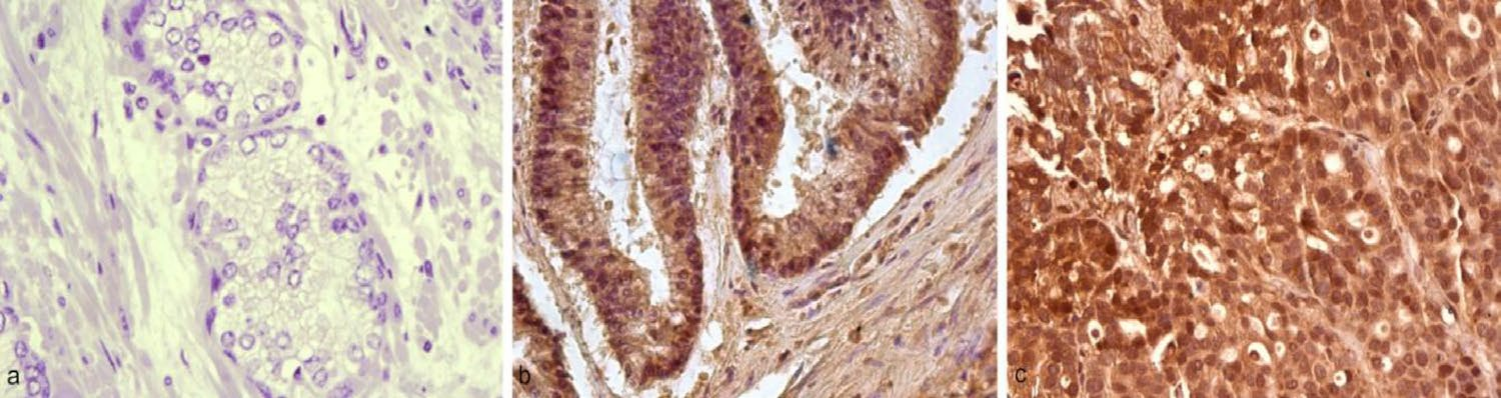
Positive and negative controls patterns for MMP14 and DCN immunoexpression. Photomicrographs demonstrating the target reactivity obtained via the indirect immunohistochemistry technique: **a** representative negative control image showing no staining in DCN and adjacent prostate tissue **b** strong cytoplasmic MMP14 staining in malignant colon tissue; **c** distinct DCN stromal immunoreactivity in malignant bladder tumor tissue. All images were captured at 400x magnification

Colon tumor tissue and bladder urothelial carcinoma—both with previously confirmed high expression of MMP14 and DCN, respectively—served as positive controls. Positive controls were included to validate the assay performance and antibody specificity. For MMP14, human colon cancer tissue was utilized as a positive control, given its well-documented high expression levels of this protein associated with extracellular matrix remodeling and tumor invasion (Devy et al. 2009). For DCN human bladder carcinoma tissue was selected as the positive control, as DCN is robustly expressed in the tumor stroma and altered urothelia during bladder malignancy progression (Iozzo 2002). All control samples were processed under identical experimental conditions as the study samples. These tissues were used to standardize primary antibody dilutions and ensure experimental reliability. For the negative control, the primary antibody incubation was omitted (Fig. 1).

Immunostaining profiles were independently evaluated by a pathologist across malignant epithelial tissue, adjacent non-tumor tissue, and the ECM. To ensure maximum consistency and minimize potential bias, all tissue samples in this study were evaluated by a single, experienced pathologist who was blinded to all clinical and prognostic data of the patients throughout the entire work.

Subcellular localization was assessed in the cytoplasmic, nuclear, and membrane compartments using a Leica DM4500 B optical microscope equipped with a DFC The staining intensity was 300FX camera at 40x magnification. The staining intensity was classified based on all fields of the available section, excluding necrotic areas, and categorized as: absent (no staining that is distinct from background signaling), weak (weak staining that is distinct from background signaling), or strong (strong staining that is distinct from background signaling) in a blinded manner, following the same protocols used for standard-of-care markers in clinical routine. A consistent exclusively cytoplasmic staining pattern for both MMP14 and DCN was observed across all malignant and adjacent non-tumor tissues. In the peritumoral stroma, immunoreactivity was confined to the extracellular matrix (ECM). A baseline signal that did not correspond to the established staining pattern for MMP14 and DCN was classified as negative. Only staining that consistently covered the cytoplasm of tumor cells was categorized as either weak or strong.

### MMP14 and DCN expression via immunofluorescence

Immunofluorescence was performed for visual validation in selected samples from the metastatic group exhibiting either weak or strong immunostaining patterns. Independent preparations were processed according to the protocol described by Pinheiro et al. (2024), using the same primary antibodies described above.

Images were captured using a Leica DM4500 B microscope and a DFC 300FX camera. Grayscale images were acquired and pseudo-colored as follows: blue for 4′,6-diamidino-2-phenylindole (DAPI) nuclear staining, yellow-green for avidin-fluorescein isothiocyanate (FITC), and red for acridine orange (AO). Image processing and overlay were performed using GIMP 2.8 (GIMP Development Team, Linux).

### Statistical analysis

Differences in MMP14 and DCN immunostaining between malignant and adjacent non-tumor tissues were assessed using the McNemar test for paired samples. Pearson’s chi-square test was employed to evaluate the distribution of immunostaining and its association with clinicopathological parameters. Kendall’s tau-b correlation was used to analyze the relationship between MMP14 and DCN immunostaining profiles (in both tumor and ECM) and prognostic factors. To identify independent predictors of clinical outcomes, a multivariate binary logistic regression model was constructed.

Data analysis was conducted using IBM^®^ SPSS^®^ Statistics for Windows, version 20.0, with a significance level of *p* < 0.05 (5%).

## Results

### Immunostaining profiles of MMP14 and DCN

**Fig. 2:**
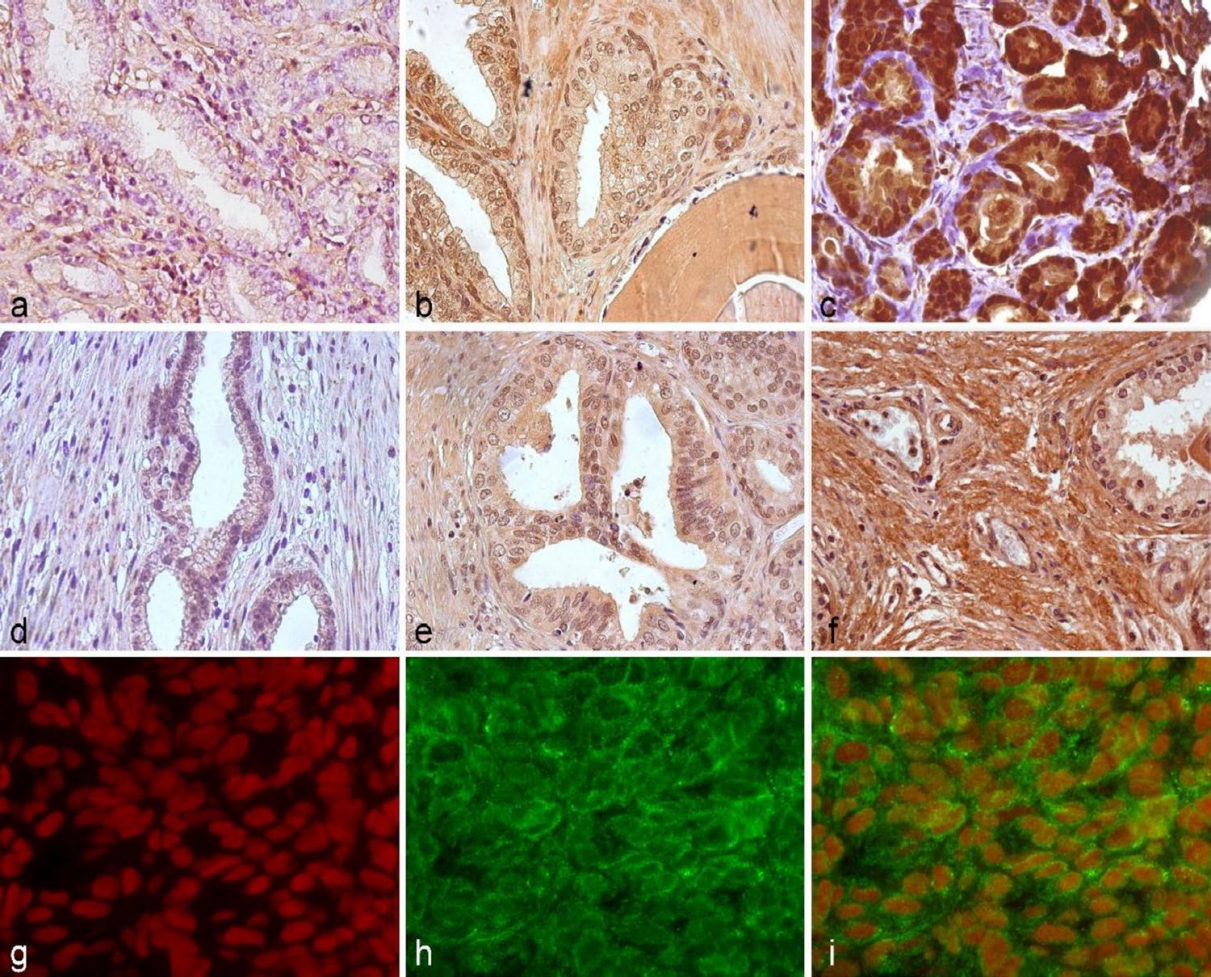
Immunostaining profiles for MMP14 obtained by indirect immunohistochemistry: **a** absence of tumor staining; **b** weak tumor staining; **c** strong tumor staining; **d** absence of ECM staining; **e** weak ECM staining; **f** strong ECM staining. MMP14 immunofluorescence technique: **g**, **h** and **i**: strong MMP14 staining in the malignant tumor in a metastatic patient sample; **g** red staining representing cell nuclei, **h** green staining representing strong staining of MMP14 protein in the malignant tumor and **i** overlay of images g and h demonstrates strong MMP14 staining within the malignant tumor. 400X. magnification

A total of 92 tissue samples were analyzed, encompassing malignant epithelium, adjacent non-tumor tissue, and the peritumoral ECM. Although the study cohort comprised 92 patients, the final sample size varied across certain analyses. Due to the absence of representative fragments of malignant tumor, adjacent non-tumor tissue, or extracellular matrix in specific tissue blocks, some patients were excluded from particular evaluations. Most risk groups exhibited weak MMP14 staining in the ECM (52/91; 57.1%) (Table 2). In the malignant tissue, weak staining was predominantly observed across the low, intermediate, and high-risk groups. In contrast, the metastatic group showed strong MMP14 expression in 60% of patients, as illustrated in Fig. 2 (a–f), which displays the protein’s immunostaining profiles in both the malignant tumor and ECM.

**Table 2:** Distribution of MMP14 immunostaining profiles among risk groups for localized (low, intermediate, and high) and metastatic disease in epithelial malignant tumor and ECM

	Tumor tissue	ECM surrounding the tumor	
MMP14	43.9% (40)	57.1% (52)	Weak staining
	38.4% (35)	32.9% (30)	Strong staining

**Fig. 3:**
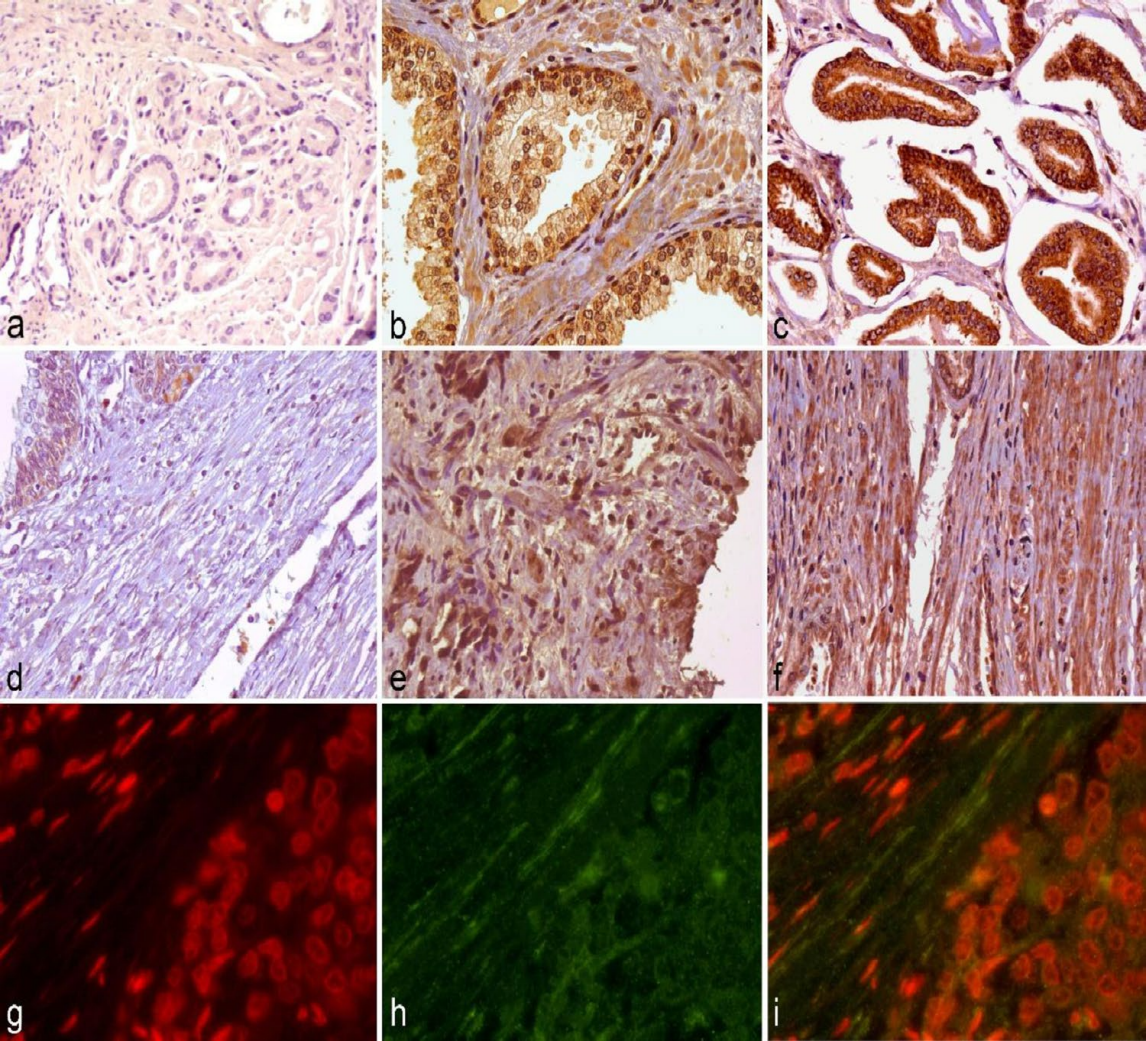
Immunostaining profiles for DCN obtained by indirect immunohistochemistry: **a** absence of tumor staining; **b** weak tumor staining; **c** strong tumor staining; **d** absence of ECM staining; **e** weak ECM staining; **f** strong ECM staining. DCN immunofluorescence technique: **g**, **h** and **i** weak DCN staining in the ECM; **g** red staining representing cell nuclei, **h** green staining representing weak DCN staining in the ECM and **i** overlay of images g and h representing weak DCN staining in the ECM. 400X magnification

Regarding DCN, strong staining was observed in the malignant tumor regions (56/87; 64.4%), whereas weak staining prevailed in the ECM (51/86; 59.3%) (Table 3). This weak ECM expression was particularly evident in the metastatic group (17/27; 63%). These patterns are exemplified in Fig. 3a–f, showing the proteoglycan immunostaining profiles in the malignant tumor and peritumoral ECM.

To visually confirm these findings, representative samples from the metastatic risk group were selected: two showing strong MMP14 expression in the tumor region and one exhibiting low DCN expression in the ECM, as illustrated in Figs. 2g–i and 3g–i.

**Table 3:** Distribution of DCN immunostaining profiles among risk groups for localized (low, intermediate, and high) and metastatic disease in epithelial malignant tumor and ECM

	Tumor Tissue	ECM surrounding the tumor	
DCN	26.4% (23)	59.3% (51)	Weak staining
	64.4% (56)	38.4% (33)	Strong staining

### MMP14 expression correlates with prognostic parameters

All parameters were evaluated for both MMP14 and DCN; however, only significant results are reported. Regarding MMP14, significant differences in immunostaining were observed between malignant epithelial tissue and adjacent non-tumor tissue (*p* < 0.001), as well as between the peritumoral ECM and adjacent non-tumor tissue (*p* < 0.001). MMP14 expression was notably higher in the epithelial tumor tissue.

High epithelial MMP14 expression correlated with the presence of metastasis (*p* = 0.003; tau-b = 0.303), consistent with the high frequency of strong expression (60%) observed in metastatic patients. Significant correlations were also found between tumor staining and both TNM staging (*p* = 0.02; tau-b = 0.356) and seminal vesicle invasion (*p* = 0.02; tau-b = 0.275) (Table 4).

In the peritumoral ECM, a significant association was identified between weak MMP14 staining and low-grade tumors (ISUP 2) (18/23; 78.3%, *p* = 0.008). Furthermore, a significant correlation was observed between MMP14 staining in ECM and the overall ISUP grade (*p* = 0.006; tau-b = -0.261) (Table 5).

**Table 4:** Results from comparisons between prognostic parameters and MMP14 staining profiles in malignant tumor tissue and ECM

MMP14	Tumor Tissue	ECM surrounding the tumor	
	*p* value of Kendall (tau-b value)	*p* value of χ2	*p* value of Kendall (tau-b value)
Metastasis	0.003 (0.303)	0.105	0.565 (− 0.059)
TNM	0.02 (0.356)	0.895	0.860 (− 0.018)
Gallbladder Invasion	0.02 (0.275)	0.055	0.572 (− 0.072)
ISUP	0.866 (0.016)	0.008	0.006 (− 0.261)

**Table 5:** Results from comparisons between prognostic parameters and DCN staining profiles in malignant tumor tissue and ECM

	ECM surrounding the tumor	
DCN	*p* value of χ2	*p* value of Kendall (tau-b value)
TNM	0.033	0.006 (− 0.296)
ISUP	0.391	0.047 (− 0.201)

### DCN expression correlates with prognostic parameters

For the DCN proteoglycan, significant differences in immunostaining profiles were observed between the malignant tissue and both the adjacent non-tumor tissue (*p* = 0.001) and the peritumoral ECM (*p* = 0.001). DCN expression was significantly higher in the malignant tumor.

Regarding prognostic parameters, a significant association was identified between strong DCN expression in the ECM and intermediate TNM stages (T2a–T3a) (*p* = 0.033). Furthermore, DCN expression in the ECM showed an inverse correlation with TNM staging (*p* = 0.006; tau-b = − 0.296).

Finally, a correlation was observed between ECM staining and ISUP grade (*p* = 0.047; tau-b = − 0.201), with 66% of patients exhibiting low DCN expression in conjunction with more aggressive tumor grades (Table 5).

### MMP14 expression as an independent predictor of metastasis

The objective was to evaluate whether the analyzed markers and other clinical parameters are associated with the presence of metastasis at diagnosis and to determine the independent contribution of these variables to this outcome.

Initial correlation analyses revealed positive associations between the presence of metastasis and MMP14 staining in tumor cells (*p* = 0.003; tau-b = 0.303), risk group (*p* < 0.001; tau-b = 0.790), PSA levels (*p* < 0.001; tau-b = 0.388), ISUP grade (*p* = 0.01; tau-b = 0.247), and seminal vesicle invasion (*p* < 0.001; tau-b = 0.501).

The contribution of these factors was further evaluated using a binary logistic regression model for metastasis prediction. The variables included in the model were: MMP14 tumor staining (dichotomized as weak/absent vs. strong), ISUP grade, and PSA levels (categorized as 0–10 ng/mL, 10–20 ng/mL, and > 20 ng/mL).

In the multivariate analysis, strong MMP14 staining was independently associated with the presence of metastasis (OR 8.01; 95% CI 1.64–39.06; *p* = 0.01), as were PSA levels above 20 ng/mL (OR 16.40; 95% CI 2.09–128.55; *p* = 0.008). Conversely, ISUP grade and age at diagnosis did not reach statistical significance in this model (Table 6).

**Table 6:** Multivariate binary logistic regression analysis of parameters associated with metastasis

Parameter	OR	IC95%	*p*
MMP14			
Absent/weak	1	ref	ref
Strong	8.01	1.64–39.06	0.001
ISUP grade			
1	1	ref	ref
2	0.232	0.024–2.22	0.204
3	0.561	0.060–5.29	0.613
4	1.94	0.246–15.37	0.528
5	1.55	0.069–34.47	0.783
PSA level			
0-10ng/mL	1	ref	ref
10-20ng/mL	4.03	0.527–30.93	0.18
> 20ng/mL	16.40	2.09–128.55	0.008

## Discussion

To our knowledge, no recent studies have investigated the tumor-suppressive role of DCN in PCa tissue samples, nor have the expression profiles of MMP14 and DCN been simultaneously evaluated across distinct prognostic subgroups. While the interplay between these molecules has been documented in other malignancies (Mao et al. 2021; Zhang et al. 2021; Li et al. 2021; Kümper et al. 2022), the present study is a pioneer in assessing their combined immunohistochemical profiles in PCa. Furthermore, our findings are particularly relevant to public health as they are derived from a Brazilian cohort treated under the Unified Health System (SUS). In low-income settings and developing countries, where access to high-cost genomic testing remains limited, the identification of reliable, low-cost immunohistochemical biomarkers is essential for improving risk stratification and clinical management.

Our findings revealed a predominance of strong epithelial MMP14 expression in the metastatic group, with 60% of these patients exhibiting high protein levels. In contrast, weak DCN staining was prevalent in the peritumoral ECM, particularly among metastatic cases, where 63% showed low proteoglycan expression. Furthermore, a significant correlation was identified between reduced ECM DCN expression and ISUP grades 4–5, with 66% of these patients presenting aggressive tumor grades. Notably, MMP14 and DCN displayed distinct expression patterns when comparing malignant epithelium to adjacent non-tumor tissues within the same patient, highlighting a clear molecular shift during tumorigenesis.

Within tumor dynamics, cancer cells undergo rapid proliferation and dissemination, necessitating survival strategies in distant tissues that rely on interactions with the ECM and its components. The tumor microenvironment is a pivotal factor in cancer progression, where chronic inflammation further contributes to tumorigenesis. In PCa, epigenetic alterations have been observed in genes encoding matrix metalloproteinases (MMPs), consequently modulating their expression within the tumor tissue. Specifically, MMP14 expression has been reported to decrease in prostate tumor tissue (Geng et al. 2020). Our data corroborates these findings, as the majority of tumor tissues in the present study exhibited weak MMP14 labeling, with the notable exception of the metastatic patient subgroup.

In this context, a recent literature review stated that prostate tissue aging promotes epigenetic changes that foster an inflammatory environment, yielding malignant cells capable of altering the composition, structure, and organization of the ECM. Furthermore, tissue aging facilitates invasion through the aberrant expression of specific matrix metalloproteinases, such as MMP14 (Di Carlo and Sorrentino 2024), as a disorganized ECM impairs cell adhesion and promotes tissue invasion and migration. In the present study, high MMP14 expression was observed in tumor cells from 60% of metastatic patients. Furthermore, high MMP14 expression was statistically associated and correlated with the presence of metastasis, consistent with the clinical profile of these patients. Similar findings have been reported in bladder cancer, where high MMP14 expression is associated with metastasis, and its downregulation has been shown to suppress cell invasion and migration (Wang et al. 2020).

A recent review stated that metalloproteinases such as MMP2, MMP7, and MMP9 are fundamental to understanding prostate tumor progression due to their roles in PCa tumorigenesis. Similarly, MMP14 is associated with pathways triggering ECM remodeling and mechanisms involving the migration of PCa-associated fibroblasts, which favor cell invasion (Samaržija 2024). These findings highlight the relevance of evaluating MMP14 as a potential therapeutic target in malignant prostate tissue. Our results corroborate this perspective, as a significant correlation was observed between epithelial MMP14 expression and seminal vesicle invasion, a clinical parameter indicative of an invasive profile and tumor progression. Conversely, a significant correlation was found between weak tumor staining and intermediate ISUP grade groups, reflecting less aggressive tumor profiles.

The young prostatic epithelium maintains its structural integrity and tissue homeostasis through cells such as fibroblasts, which regulate immune functions and aid in the secretion of molecules such as MMPs, recognized for their role in remodeling the ECM (Di Carlo and Sorrentino 2024). In another study, they found a positive correlation between StromalScore and the expression of MMP14 in prostate tumor tissues, showing that the joint activity of fibroblasts with MMP14 can influence tumor behavior, affecting its progression (Li et al. 2021). In the present study, the categorized prognostic risk groups exhibited a weak MMP14 staining profile in the tumor-surrounding ECM. Additionally, a significant association and correlation were observed between weak MMP14 expression and low-grade tumors (ISUP grade group 2), suggesting that reduced MMP14 expression is linked to a less aggressive clinical behavior in prostate cancer.

Another critical factor supporting tumor survival is neoangiogenesis, which ensures adequate nutrient supply to the expanding tumor mass. A recent study demonstrated that MMP14 plays a key role in regulating angiogenesis within malignant tissues, thereby promoting metastasis; notably, the loss of MMP14 function has been shown to decrease tumor growth in melanoma (Kümper et al. 2022). Our findings reveal that patients in the low- and intermediate-risk groups exhibited absent or weak MMP14 expression compared to the metastatic group. Consequently, it is plausible that this reduced expression limits neoangiogenesis, potentially hindering the metastatic cascade in these specific patient subgroups.

Previous research has demonstrated that MMP14 expression differs significantly between malignant epithelial tissues and adjacent non-tumor tissues (Li et al. 2021). In the present cohort, a significant discrepancy in MMP14 labeling was also observed when comparing both epithelial tumor tissue and the surrounding ECM against adjacent non-tumor counterparts. These findings underscore the differential expression of MMP14 within the tumor mass and its microenvironment, further reinforcing its potential as a robust diagnostic or prognostic tumor marker.

It is widely recognized that prognostic factors help estimate a tumor’s degree of aggressiveness. Among these, the ISUP grade stands out, used to classify patients into different risk groups (Srigley et al. 2019), and TNM staging, which assesses the anatomical extent of the tumor and identifies the presence or absence of metastases (Brierley et al. 2021). Although the present study found no significant association between DCN expression in tumor cells and prognostic parameters, a significant correlation was observed between strong stromal DCN expression and intermediate pathological staging (pT2a–pT3a). Furthermore, a significant correlation was identified regarding ISUP grade groups 4–5, where 66% of these high-grade patients exhibited low DCN expression. These findings underscore that alterations in the ECM components surrounding the tumor may play a pivotal role in the prognosis of prostate cancer, highlighting the importance of the microenvironment over purely epithelial protein expression.

Proteoglycans such as DCN are component molecules of ECM and are present in the tumor microenvironment, acting in processes that determine metastasis, dormancy, or death of the primary tumor (Iozzo and Gubbiotti 2018). A DCN deficient tumor microenvironment has been observed to favor the induction of epithelial-mesenchymal transition (EMT) and induce metastasis in colorectal cancer (Li et al. 2021). In another recent study, DCN was found to negatively regulate genes associated with lymphatic vessel development in breast tumor tissue, acting as a tumor suppressor. Its high expression was indicative of a good prognosis in breast cancer (Mondal et al. 2024). Within this context, previous studies have demonstrated that low DCN levels in the liver facilitate the development of colon carcinoma metastases, suggesting a potential antitumor role for this proteoglycan. Conversely, high DCN expression has been shown to inhibit the formation of metastatic liver tumors (Reszegi et al. 2020). Consistent with these reports, the present study observed weak DCN labeling in the ECM surrounding the tumors of metastatic and high-risk patients. This reduced expression may foster an environment conducive to the invasive process in PCa.

Finally, several studies have underscored the multifaceted role of DCN in cancer regulation: it inhibits metastasis and invasion while promoting cell adhesion in bladder cancer (Chen et al. 2022), suppresses proliferation and inactivates growth signaling pathways in thyroid carcinoma (Liu et al. 2024) and impairs proliferative pathways while inducing apoptosis in PCa (Hu et al. 2009). Overall, our findings are consistent with the existing literature, suggesting that the reduced presence of the DCN proteoglycan within the tumor microenvironment may facilitate disease progression toward more aggressive stages and subsequent dissemination.

This study has both strengths and limitations that warrant consideration. A primary limitation is the relatively small sample size in the low-risk group. This reflects the patient demographic of our referral center, which predominantly manages advanced-stage cases within the Brazilian Unified Health System (SUS), accurately mirroring the clinical profile of PCa in the southern Brazilian population. Due to the retrospective nature of this cohort and the unavailability of long-term post-operative follow-up records for these patients, tracking survival timelines was not feasible. However, our primary objective was to evaluate the association of these markers with established clinicopathological indicators of immediate tumor aggressiveness and advanced disease at the time of diagnosis. Conversely, the study’s strengths include a rigorous histopathological evaluation performed by a specialist in IHC, ensuring high-quality interpretation of tumor, adjacent non-tumor, and peritumoral ECM. Furthermore, the use of IHC as the gold standard, combined with the availability of comprehensive clinicopathological data which allowed for precise risk stratification by our urologists following standardized clinical protocols significantly enhances the robustness and clinical relevance of our findings. Finally, in Brazil, there is a clinical gap that includes failures in screening for active surveillance, in addition to limitations in the use of PSA, and the challenges of late diagnosis in the SUS, which justifies the relevance of researches such as those of the present study, aiming at the incorporation of easily applicable and low-cost markers.

## Conclusion

In summary, our results demonstrate that MMP14 and DCN are differentially expressed between malignant tumor and adjacent non-tumor tissue. These markers showed associations with key prognostic parameters, most notably the presence of metastasis, which was independently predicted by high MMP14 expression in tumor cells. These findings suggest MMP14 and DCN as promising biomarkers for assessing prognosis and risk of tumor dissemination in PCa. Ultimately, a deeper understanding of extracellular matrix components and their remodeling is essential to enhance prognostic accuracy and guide more personalized and effective therapeutic strategies in prostate carcinogenesis.

## Data Availability

No datasets were generated or analysed during the current study.
